# Integrated Metagenomic and LC–MS/MS Analysis Reveals the Biogenic Amine-Producing Strains of Two Typical Chinese Traditional Fish Products: Fermented Mandarin Fish (*Siniperca chuatsi*) and Semi-Dried Yellow Croaker (*Larimichthys crocea*)

**DOI:** 10.3390/foods14061016

**Published:** 2025-03-17

**Authors:** Xuan Zhang, Hai Chi, Di Peng, Mei Jiang, Cuihua Wang, Haiyan Zhang, Wei Kang, Lei Li

**Affiliations:** 1 East China Sea Fisheries Research Institute, Chinese Academy of Fishery Sciences, Shanghai 200090, China; zhangxuan@ecsf.ac.cn (X.Z.); pengdi@ecsf.ac.cn (D.P.); jiangmei@ecsf.ac.cn (M.J.); wangch@ecsf.ac.cn (C.W.); zhanghy@ecsf.ac.cn (H.Z.); kangw@ecsf.ac.cn (W.K.); 2 Key Laboratory of Protection and Utilization of Aquatic Germplasm Resource, Liaoning Ocean and Fisheries Science Research Institute, Dalian 116023, China; chih@ecsf.ac.cn

**Keywords:** metagenomes, microbial community, biogenic amine-producing bacteria, biogenic amines, fish product

## Abstract

Two typical fish products—fermented mandarin fish and semi-dried yellow croaker—are associated with biogenic amines (BAs), which are harmful to human health. The objective of this study was to investigate the bacterial ecology of the two fish products and to determine their capacity for producing BAs. Putrescine and cadaverine were major BAs detected in the fish products. Concentrations of BAs were significantly corrected with microbial count (*p* < 0.05). BA-producing isolates (33) in the two fish products were all multiple BA producers. Several of them, including *Lactobacillus sakei*, *Bacillus cereus* and *Hafnia alvei* isolated from fermented mandarin fish, as well as *Shewanella baltica*, *Aeromonas veronii*, and *Photobacterium phosphoreum* isolated from semi-dried yellow croaker, showed remarkable BA-producing capacity. *Hafnia alvei* produced the greatest abundance of putrescine, cadaverine, tyramine and 2-phenylethylamine. *Lactobacillus sakei* mainly produced tryptamine and putrescine. *Photobacterium phosphoreum* showed the strongest histamine-producing capacity.

## 1. Introduction

Fermented mandarin fish (*Siniperca chuatsi*) and semi-dried yellow croaker (*Pseudosciaena crocea*) are typical traditional Chinese fish products. Due to their abundance of nutrients and unique flavors, these two fish products have gained increased popularity among consumers in China [1,2]. The production of fermented mandarin fish involves marinating mandarin fish in brine with a low salt content of 4–8% (*w*/*v*), which is mixed with spices such as chili, anise, fennel, ginger and Chinese prickly ash, and then subjecting it to spontaneous fermentation for 7–10 days. Semi-dried yellow croaker is typically prepared by salting fresh large yellow croaker in brine with a low salt content of 4–8% (*w*/*v*) and white liquor, followed by sun-drying or oven-drying under natural conditions until the muscle tissues attain a moisture content of 40–60%. The pH of commercially available semi-dried yellow croaker varies from 6.08 to 6.97. However, the traditional production process is uncontrollable, with a complex microbial structure that readily produces substances which are harmful to humans, such as biogenic amines (BAs). Thus, high levels of BAs are commonly observed as defects in traditional meat products [3].

BAs are low molecular weight nitrogenous compounds, produced from free amino acids by decarboxylation. These BAs negatively affect the quality of fish products. In addition, as BAs are associated with vasoactive and psychoactive properties, their presence could be considered a potential health risk for some consumers [4]. What is worse, according to a previous report, BAs are difficult to eliminate once they are formed [5,6]. Therefore, it is necessary to inhibit the production of BAs in food during the processing and storage processes.

BAs are influenced by the bacterial community, amino acids, environmental factors, processing methods, and storage conditions of food [7]. Generally, the predominant genera found in fish products are *Pseudomonas*, *Vibrio* and *Shewanella* [6,8]. Strong BA production bacteria are commonly associated with *Photobacterium phosphoreum*, *Hafnia alvei*, *Luminobacterium* spp., *Photobacterium phosphoreum* and *Morganella morganii* [9]. These isolates always possess the capabilities for multiple BA production. However, the bacteria that contribute to BA accumulation in the two typical traditional Chinese fish products are not clear. Thus, the objective of the present research was to investigate the BAs and bacterial community in the two typical species of fish products using liquid chromatography–tandem mass spectrometry (LC–MS/MS) and high-throughput sequencing. Since the production and sales of fermented mandarin fish are primarily concentrated in Huangshan and Shanghai, while the main production areas of semi-dried yellow croaker are Ningde and Zhoushan, the two fish products from these regions have been selected for this research. The isolation, characterization and identification of BA-producing bacteria were also carried out in order to study their roles in BA production. To the best of our knowledge, this is the first systematic study of BA-producing bacteria in these two types of aquatic products.

## 2. Materials and Methods

### 2.1. Collection of Samples

A total of 20 specimens of fish products were randomly obtained from the local markets in Shanghai. The examined fish products were divided into 2 groups: (a) fermented mandarin fish (*Siniperca chuatsi*) samples (5 from Huangshan and 5 from Shanghai), and (b) semi-dried yellow croaker (*Pseudosciaena crocea*) samples (5 from Ningde and 5 from Zhoushan). To ensure the regional representativeness of the fish samples collected from each city, the samples originating from the same city were combined and promptly utilized for BA analysis, viable cell counting and microbial assay. All the fish products were transported immediately to the laboratory using a portable refrigerator for viable cell counts and microbial assays. The rest of the fish samples were frozen at −80 °C for chemical analysis and DNA extraction.

### 2.2. Biogenic Amine Analysis

BAs, including cadaverine (CAD), putrescine (PUT), tyramine (TYR), 2-phenylethylamine (2-PHE), histamine (HIS) and tryptamine (TRY), were determined using HPLC–MS/MS [10]. In brief, BAs were extracted twice from 2 g fish samples with 40 mL of 5% HClO_4_. The extraction was then adjusted to pH 3 with NaOH solution and was diluted with 0.5% HClO_4_ solution. A total of 2 mL of n–hexane was added for cleaning up. Then 0.25 mL of the purified solution was diluted by 0.75 mL of acetonitrile. The final mixture was analyzed by liquid chromatography–tandem mass spectrometry (LC–MS/MS) using a Shimadzu LC–30AD solvent delivery unit (Shimadzu, Kyoto, Japan) connected to a 5500 QTRAP MS (AB SCIEX, Framingham, MA, USA). The target compounds were separated at 35 °C on a Waters XBridge^®^ HILIC column (150 mm × 2.1 mm, 3.5 μm, Waters Technologies Ltd., Drinagh, Ireland). The limits of detection (LODs) were in the range of 0.1–1.0 mg/kg. An intermediate level of the standard solution was repeatedly detected every ten fish samples to calculate the reproducibility of the results. All the fish samples were examined with three replicates.

### 2.3. Total Visible Count and Microbial Community Analysis

The total visible count (TVC) was detected using plate count agar (PCA) medium (Sangon, Shanghai, China). A 25.0 g fish sample was weighed and homogenized with 225 mL of 0.85% (*w*/*v*) NaCl solution. The solution was then gradient diluted and spread on PCA medium. The TVC was determined after 2 d of incubation at 37 °C.

Total community genomic DNA extraction was performed using a Mag-Bind Soil DNA Kit (Omega, M5635-02, Norwalk, CA, USA), following the manufacturer’s instructions. The concentration of the DNA was measured using a Qubit 4.0 fluorometer (Thermo, Waltham, MA, USA) to ensure that adequate amounts of high-quality genomic DNA had been extracted. The V3–V4 hypervariable region of the bacterial 16S rRNA gene was amplified using the primers 341F (CCTACGGGNGGCWGCAG) and 805R (GACTACHVGGGTATCTAATCC). The PCR reactions were set up using 2 × Hieff^®^ Robust PCR Master Mix (Yeasen, 10105ES03, Shanghai, China) and the PCR products were purified with Hieff NGS™ DNA Selection Beads (Yeasen, 10105ES03, Shanghai China). Sequencing was performed with the Illumina MiSeq sequencing system (Illumina MiSeq, San Diego, CA, USA). The Illumina readings were assembled using PEAR software (version 0.9.8). Effective tags were classified as operational taxonomic units (OTUs) of 97% similarity using Usearch software (version 11.0.667). The most abundant sequence was selected as the OTU representative sequence and classified taxonomically with the Ribosomal Database Project (RDP) Classifier. Alpha Diversity was estimated with Mothur (version 3.8.31) to reflect microbial community diversity in the two fish products.

### 2.4. Bacterial Diversity Analysis by the Culture-Dependent Method

#### 2.4.1. Screening of BA-Producing Bacteria

The BA decarboxylase activity of bacteria was isolated according to the screening plate method described by Deng et al. [11] with slight modifications. The fish sample (10 g) was diluted with 90 mL of 0.85% (*w*/*v*) NaCl solution and homogenized. The homogenate was then serially diluted and spread on Niven’s agar with a pH of 5.3 (0.5% yeast extract, 0.5% peptone, 2% agar, 0.25% phenylalanine, 0.25% tryptophan, 0.25% lysine, 0.25% tyrosine, 0.25% arginine, 0.25% histidine, 0.25% ornithine hydrochloride, 0.5% sodium chloride, 0.1% calcium carbonate, 0.005% pyridoxal 5′-phosphate and 0.006% bromocresol violet). Strains on Niven’s agar plates were incubated at 37 °C for 48 h. Colonies with various morphologies were selected and the isolates were purified four times. The pure colonies were inoculated and stored at −80 °C in Luria–Bertani (LB) liquid medium containing glycerol (50%, *v*/*v*).

#### 2.4.2. Identification of BA-Producing Bacteria

DNA extraction of BA-producing bacteria was performed according to the previously described method of Wang et al. [12]. The 16S rRNA gene sequencing method was used to identify each BA-producing bacterium. A 25 μL amplification system, with a DNA template of (1 μL), 27F: AGAGTTTGATCMTGGCTCAG (1 μL), 1492R: GGTTACCTTGTTACGACTT (1 μL), Tac Master Enzyme (12.5 μL) and sterile water (9.5 μL) was prepared for an ordinary PCR reaction under the following conditions: 95 °C for 5 min, followed by 30 cycles (94 °C for 30 s, 57 °C for 30 s and 72 °C for 90 s), with a final extension at 72 °C for 10 min. The PCR-amplified products were sequenced using 1.5% agarose gel electrophoresis by Sangon Biotech (Shanghai, China).

#### 2.4.3. Determination of BA-Producing Capacity of Bacteria

The strains of BA-producing bacteria were cultured in the LB liquid media containing 0.25% ornithine hydrochloride, phenylalanine, tryptophan, lysine, tyrosine, arginine and histidine for 48 h at 30 °C. Concentrations of BAs in bacterial cultures were detected with LC–MS/MS according to the previous method [10].

### 2.5. Statistical Analysis

All experiments were analyzed in triplicate independently. The BA contents and cell counts were expressed as means ± standard deviation. The statistical analysis was carried out using SPSS 16.0 software (*p* < 0.05, SPSS Inc., Chicago, IL, USA). The analysis of correlation was carried out using Pearson’s correlation coefficient. The analysis of BA contents and cell counts in different fish samples was statistically performed using the Wilcoxon test.

## 3. Results and Discussion

### 3.1. BA Levels and Microbial Counts in Fish Products

The levels of BAs obtained by LC–MS/MS and microbial counts in fish products purchased from the four cities are listed in Table 1. The results indicated that the most abundant BA detected in fish product samples was PUT (56.84 ± 5.35 mg/kg), followed by CAD (36.90 ± 2.55 mg/kg). In addition, CAD and HIS were detected in all the fish samples. However, TRY (0.91 ± 0.05 mg/kg) presented the lowest level and occurrence in the two fish products. In the case of fermented mandarin fish, five BAs were detected in both the Huangshan and Shanghai samples, with the exception of TRY. These results for BAs in fish products were in agreement with previous studies [6,13]. For the contribution of each BA to total contents in these fish samples, the fermented mandarin fish samples, which showed higher levels of BAs, were characterized by a high contribution of PUT (52.1%) to the total BA content, followed by CAD (32.5%). In semi-dried yellow croaker samples, CAD (36.1%) represented the most prevalent BA in relation to total levels. The amounts of BAs and microbial counts in fermented mandarin fish samples were significantly higher than those in semi-dried yellow croaker samples (*p* < 0.05).

Overall, the profile and level of BAs differed among the fish species and regions. The correlations were carried out using Pearson correlation coefficients. The BAs showed a significant positive correlation with microbial count (*p* < 0.05). This result suggested that substrates and microbial communities in different fish products could contribute to the high levels of BAs in fermented mandarin fish samples [3,7]. In this study, safe levels of HIS and TYR in the two fish products were observed [5]. According to previous studies, PUT and CAD can intensify the toxic effects of HIS and TYR, leading to harmful effects on human health [3,9]. Therefore, high levels of PUT and CAD in fermented mandarin fish products deserve more attention, as the amounts of BAs could increase during storage. BA accumulation depends on the substrates, microbiota and environmental conditions (such as temperature, salinity, humidity and storage duration) present during processing and storage. BAs are difficult to eliminate once they are formed. Therefore, approaches including using appropriate starter culture, appropriate salinity, low temperature and high pressure could be used to reduce the health hazard of BA formation in raw and processed food.

### 3.2. Alpha Diversity in Fish Products

The diversity indices and coverage of fish samples from four different regions were detected (Table 2). The coverage of all fish samples exceeded 0.998, indicating that sufficient microorganisms were detected in the samples. A total of 345,009 effective sequences were clustered into 1859 OTUs. In general, the Chao and Ace values are used to estimate the richness of bacteria in samples. The Shannon and Simpson indices reflect the diversity of bacteria [14,15]. The Chao and Ace values in semi-dried yellow croaker were significantly higher than those in fermented mandarin fish (*p* < 0.01), as were the Shannon and Simpson indices. The results indicated that the semi-dried yellow croaker samples exhibited higher richness and diversity of bacteria compared to the fermented mandarin fish. However, the differences in bacterial richness and diversity between regions for the same fish products were not statistically significant (*p* > 0.05). These results were similar to previous reports [2,8].

**Figure 1:**
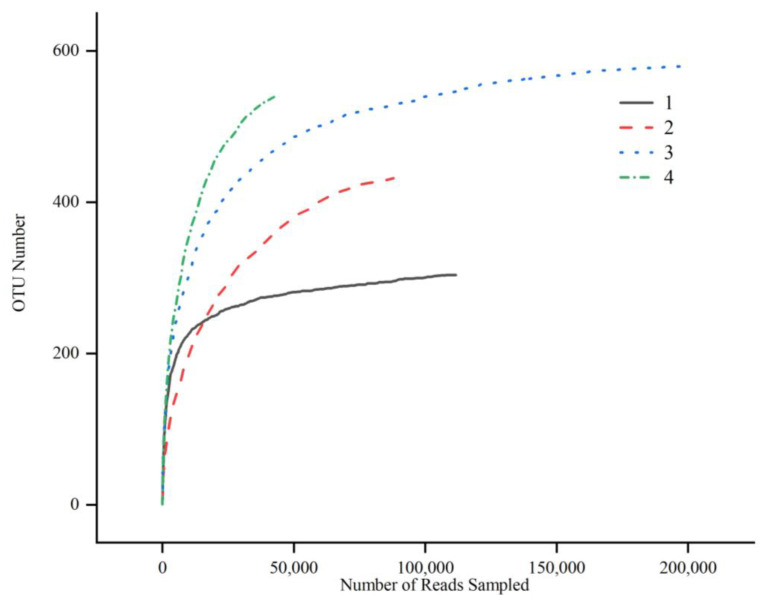
Rarefaction curves of microorganisms in fish samples. (1) Fermented mandarin fish from Shanghai, (2) fermented mandarin fish from Huangshan, (3) semi-dried yellow croaker from Ningde, and (4) semi-dried yellow croaker from Zhoushan.

The rarefaction curves of microorganisms in fish samples from different regions are shown in Figure 1. The curve was established using sequencing data and the corresponding OTU numbers. The curves for semi-dried yellow croaker were higher than those of fermented mandarin fish, implying higher bacterial richness in semi-dried yellow croaker samples. All the rarefaction curves of microorganisms tended to be steady, which reflected the adequacy of the amounts of sequencing data.

### 3.3. Composition of Bacterial Communities in Fish Products

**Figure 2:**
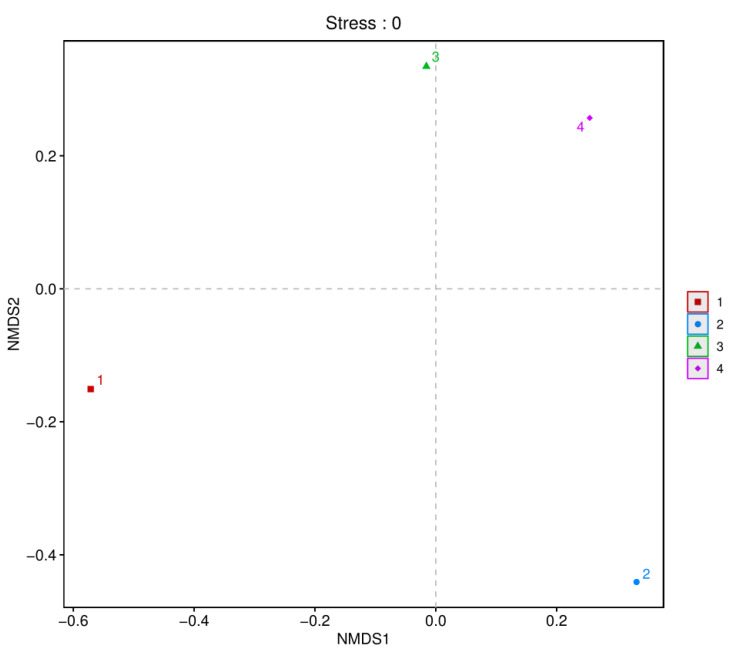
Beta diversity analysis of bacterial communities in fish products at the OTU level. (1) Fermented mandarin fish from Shanghai, (2) fermented mandarin fish from Huangshan, (3) semi-dried yellow croaker from Ningde, and (4) semi-dried yellow croaker from Zhoushan.

Beta diversity analysis is commonly used to describe the diversity of species composition. The dimensionality reduction technique of non-metric multidimensional scaling (NMDS) can be used to analyze the similarity or dissimilarity between microbial samples. The distribution of samples along continuous sorting axes (NMDS axes) can reveal general patterns in data changes. This study used NMDS to identify similar or dissimilar microbial communities between the two fish products from different regions. As shown in Figure 2, beta diversity analysis was performed based on OUT level. Significant differences in microbial communities were observed between the two fish products. The compositions of the bacterial communities in fermented mandarin fish samples from various regions differed, whereas semi-dried yellow croaker samples from different regions clustered more closely, demonstrating similarities in their bacterial communities.

**Figure 3:**
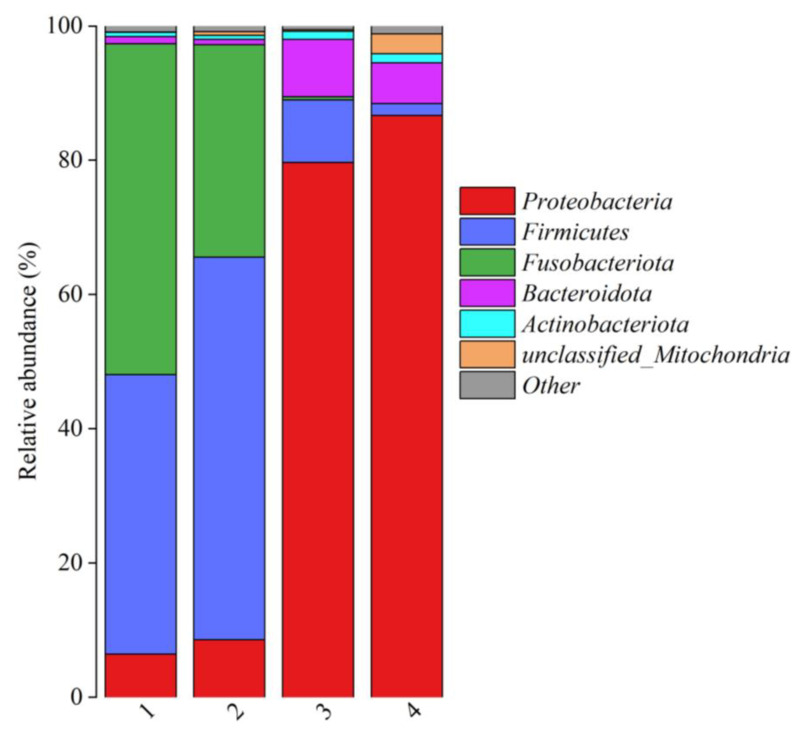
Relative abundances of bacterial communities at the phylum level among fish products from different regions. (1) Fermented mandarin fish from Shanghai, (2) fermented mandarin fish from Huangshan, (3) semi-dried yellow croaker from Ningde, and (4) semi-dried yellow croaker from Zhoushan.

The relative abundances of bacterial communities at the phylum level in fish products from different regions are shown in Figure 3. Sequencing revealed that six bacteria phyla—*Proteobacteria*, *Firmicutes*, *Fusobacteriota*, *Bacteroidota*, *Actinobacteriota*, and *unclassified_Mitochondria*—were detected in the fish product samples. Although similar microorganisms were observed in fish product samples, notable differences emerged in their bacterial compositions. *Firmicutes*, *Fusobacteriota* and *Proteobacteria* were the dominant phyla in fermented mandarin fish samples. This result was similar to previous studies [2,15]. It has been reported that *Firmicutes* and *Proteobacteria* are common phyla, accounting for 90% of identified bacterial gene sequences [16]. *Proteobacteria*, *Firmicutes* and *Bacteroidota* were the major phyla in the semi-dried yellow croaker samples. It has been reported that *Proteobacteria* and *Firmicutes* are usually observed to be the major communities in marine fish [1,9]. The microorganisms detected in the samples likely originated from the raw materials employed in the fermentation process and could be considered as environmental microorganisms from the water, soil, and air. According to previous studies, amino acids and lipid metabolisms exhibit a close correlation with the presence of *Proteobacteria*, whereas carbohydrate metabolism is intimately associated with the presence of *Firmicutes* [17].

**Figure 4:**
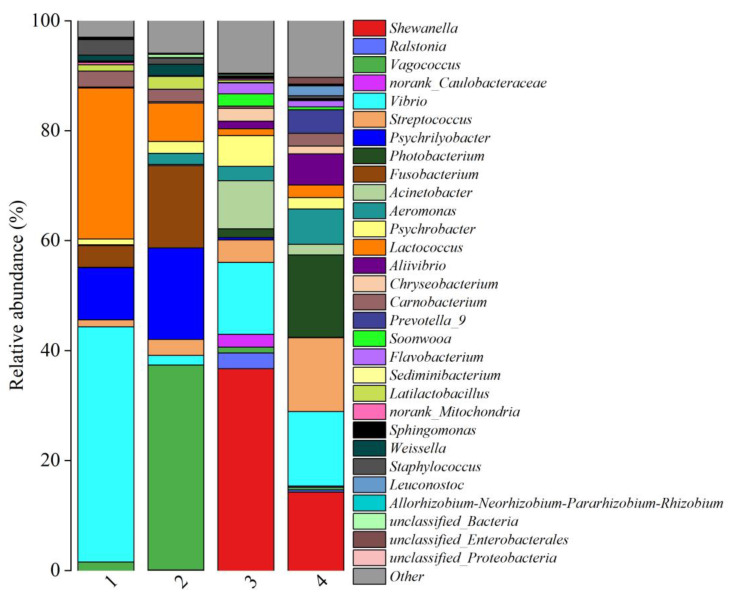
Relative abundance of bacterial communities at the genus level among fish products from different regions. (1) Fermented mandarin fish from Shanghai, (2) fermented mandarin fish from Huangshan, (3) semi-dried yellow croaker from Ningde, and (4) semi-dried yellow croaker from Zhoushan.

Figure 4 shows the relative abundances of bacterial communities at the genus level in the two fish products from different regions, and 30 bacteria genera were obtained. The microbial communities at the genus level were found to be similar within the same fish product from different regions, with notable differences between the two species of fish products. Moreover, the bacterial abundances were different in fish products purchased from different regions. The relative abundances of *Vibrio*, *Vagococcus*, *Lactococcus*, *Psychrilyobacter*, *Fusobacterium*, *Carnobacterium*, *Streptococcus*, *Staphylococcus*, *Latilactobacillus*, *Psychrobacter*, *Weissella* and *Aeromonas* exceeded 2% in fermented mandarin fish samples. This result was similar to previous studies [2,8]. For the semi-dried yellow croaker samples, relative abundances of *Shewanella*, *Vibrio*, *Streptococcus*, *Photobacterium*, *Acinetobacter*, *Aeromonas*, *Psychrobacter*, *Aliivibrio*, *Prevotella_9*, *Chryseobacterium*, *Lactococcus*, *Ralstonia*, *Soonwooa*, *Carnobacterium* and *norank_Caulobacteraceae* were over 2%. In addition, most of these bacteria are halotolerant or moderately halophilic, which are commonly observed in cured fish products [18]. In the present study, high abundances of *Vibrio* were found in all the fish samples, which is commonly identified as the primary cause of food spoilage. Meanwhile, it was reported that *Vibrio* produced BAs in fish and fish products [11,19]. The microbial genera of *Shewanella*, *Photobacterium*, *Streptococcus* and *Lactococcus* have been widely found in seafood. Due to their high potential for causing spoilage, these genera are frequently associated with food spoilage [20]. Moreover, these genera are considered to be the primary contributors to the formation of BAs from amino acids in fish. It is reported that PUT and CAD are the main BAs produced by *Shewanella baltica*. The *Photobacterium* sp. could produce 1000 mg/kg of HIS in fish [21]. *Streptococcus* and *Lactococcus* are intestinal flora of fish which are positively related to concentrations of 2-PHE, TYR and PUT [22,23]. *Vagococcus* is considered to be one of the pathogens present in aquaculture [24]. *Psychrilyobacter* could promote the hydrolysis of alanine and produce BAs and flavor substances in fermented mandarin fish [2].

### 3.4. Screening and Identification of BA-Producing Bacteria

In order to screen the BA-producing bacteria in fish products, the BA-producing bacteria screening plate method was applied according to Deng et al. [11]. After four repetitions of purification, a total of 33 BA-producing bacteria strains were screened in the two fish products (Table 3). Different bacteria species can exhibit the same BA-producing characteristics and similar phenotypic characteristics [25]. Therefore, it is necessary to use more reliable and precise identification methods to describe BA-producing bacteria in fish products. The 16S rRNA gene-sequencing analysis was applied to identify decarboxylase-positive isolates from the fish products.

The result showed that seven and nine BA-producing bacteria strains were screened in fermented mandarin fish from Shanghai and Huangshan, respectively. BA-producing bacteria included *Lactobacillus sakei* (4), *Bacillus cereus* (3), *Hafnia alvei* (3), *Psychrobacter faecalis* (2), *Vagococcus fluvialis* (1), *Aeromonas veronii* (1), *Staphylococcus caprae* (1) and *Lactococcus garvieae* (1). The strain with the largest bacterial count in fermented mandarin fish belonged to the species *Lactobacillus sakei* (4), followed by *Bacillus cereus* (3) and *Hafnia alvei* (3). Regarding semi-dried yellow croaker samples, seven and ten strains of BA-producing bacteria were isolated from Ningde and Zhoushan, respectively. The bacteria species isolated in the samples included *Shewanella baltica* (6), *Aeromonas veronii* (3) *Ralstonia pickettii* (1), *Acinetobacter johnsonii* (1), *Psychrobacter faecalis* (1), *Streptococcus parauberis* (1), *Vibrio aphrogenes* (1), *Photobacterium phosphoreum* (1), *Prevotella 9* sp. (1) and *Aliivibrio salmonicida* (1). The most abundant BA-producing species in semi-dried yellow croaker samples was *Shewanella baltica* (6), followed by *Aeromonas veronii* (3). These results revealed that the abundances and species of BA-producing bacteria were different in various fish products. Moreover, the variability of microbial communities was linked to differences in fermentation methods between regions. Most of the bacteria species isolated in our study have often been reported in fermented products [8,26]. However, different strain species exhibit various capacities for BA production, owing to the presence of diverse amino acid decarboxylases within these bacteria [27,28,29]. The presence of specific genes encoding decarboxylases in bacteria affects the production of BAs.

### 3.5. Aminogenic Capacity of BA-Producing Bacteria

**Figure 5:**
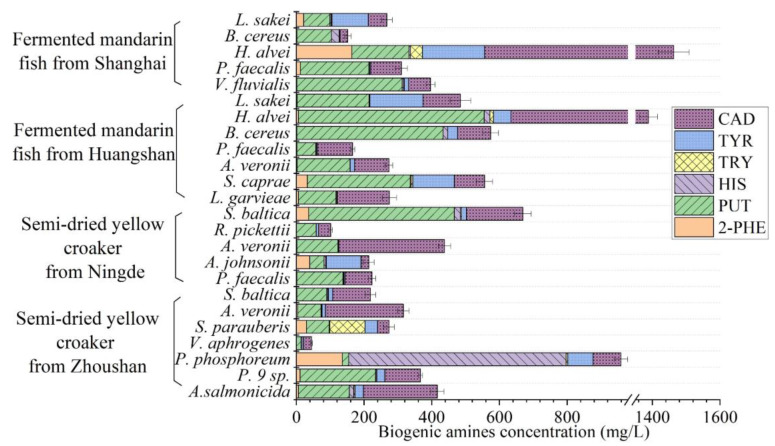
BA production of bacteria isolated from the two fish products. CAD—cadaverine; TYR—tyramine; TRY—tryptamine; HIS—histamine; PUT—putrescine; and 2-PHE—2-phenylethylamine.

The capacity of each BA-producing bacteria species was assessed by the presence of BAs in the LB liquid media. In this study, all the microorganisms were capable of producing multiple BAs, albeit with significantly varying capacities (Figure 5). The largest amount of BA produced by bacteria was PUT, ranging from 13.33 to 549.43 mg/L (mean = 170.83 mg/L), and CAD, ranging from 22.95 to 907.60 mg/L (mean = 165.67 mg/L), followed by TYR ranging from 1.37 to 183.66 mg/L (mean = 41.81 mg/L) and HIS ranging from ND to 642.54 mg/L (mean = 34.88 mg/L). Among all the 33 strains, 33 produced PUT, CAD and TYR, 32 produced HIS, 31 produced 2-PHE and 28 produced TRY.

In fermented mandarin fish, *Hafnia alvei* was able to form up to 907.60 mg/L of CAD, 549.43 mg/L of PUT, 183.66 mg/L of TYR, 16.54 mg/L of HIS, 164.13 mg/L of 2-PHE and 1463.06 mg/L of Bas in total. In this study, *Hafnia alvei* produced the greatest abundances of CAD, PUT, TYR and 2-PHE, and was also detected as a HIS producer in food. Lavizzari et al. [30] reported that this bacterium could produce more than 1000 mg/L of HIS. Ding et al. [26] described its capability of generating CAD, PUT and HIS. Li et al. [31] reported that *Hafnia alvei* produced abundant PUT and CAD in turbot flesh.

In the present study, *Lactobacillus sakei* was the most frequently identified bacterium in fermented mandarin fish. It is generally reported that in aquatic products, particularly fermented fish, *Lactobacillus sakei* exhibits a remarkable capacity for producing TYR and PUT [32]. In recent years, *Lactobacillus sakei* was used to inhibit BA formation in fish products during processing [27,33,34].

*Bacillus cereus* produced a high level of PUT (432.49 mg/L) and HIS (22.98 mg/L) isolated from fermented mandarin fish. *Bacillus cereus* is usually regarded as a saprophytic bacterium and is frequently detected in fermented food products as a BAs producer [35]. In addition, because of a strong capability for catabolizing meat components, *Bacillus cereus* can also produce flavor-rich metabolites during fermentation [36]. The isolates of *Vagococcus fluvialis*, *Psychrobacter faecalis*, *Aeromonas veronii*, *Staphylococcus caprae* and *Lactococcus garvieae* mainly produced PUT and CAD in this study. These BA-producing bacteria might also contribute to the unique flavor formation of fermented mandarin fish [2,8].

For semi-dried yellow croaker, *Shewanella baltica* was the most frequently identified bacterium in the present study. This species has often been reported as a powerful PUT-producing microorganism which is present in aquatic products [37]. Indeed, PUT- and CAD-producing strains of *Shewanella baltica* have been isolated in several seafood species, such as large yellow croaker, mussel, shrimp, golden pomfret and horse mackerel [38]. Bao et al. [39] reported that up to more than 1000 mg/L of PUT was produced by these bacteria.

*Photobacterium phosphoreum* was the most powerful HIS (642.54 mg/L) producer, which aligns with previous studies [37,40]. *Photobacterium* is widespread in marine waters and is often isolated from seafood. Bjornsdottir-Butler et al. [41] reported that *Photobacterium phosphoreum* produced the largest amount of HIS among all the *Photobacterium* species studied. In our study, the *Photobacterium phosphoreum* isolated from semi-dried yellow croaker could produce a high level of 2-PHE. Therefore, strains identified from different fish showed differences in BA-producing capacity.

*Acinetobacter johnsonii* is commonly isolated from aquatic products such as carp and sea bream [42]. In this study, *Acinetobacter johnsonii* present strong TYR (102.58 mg/L)-generating capability. According to Wang et al. [12], this species, isolated from grass carp, had moderate TYR-producing capability. *Streptococcus parauberis* produced up to 103.81 mg/L of TRY and also contributed to the generation of amino acids and flavor in fermented soybean products [43]. Other isolates identified in semi-dried yellow croaker, including *Ralstonia pickettii*, *Aeromonas veronii*, *Psychrobacter faecalis*, *Vibrio aphrogenes*, *Prevotella 9* sp. and *Aliivibrio salmonicida*, were all found to be producers of CAD and PUT, as reported by other authors [26,37].

These findings suggest that the BA-producing ability of these bacteria in the two fish products might pose a risk of BA accumulation during storage. However, BA-producing microorganisms were probably not the primary causes of the high levels of BAs in fish products, as the presence of BAs is also influenced by food substrates and environmental factors (such as temperature, humidity and storage duration) [4,23]. Further research should focus on the influence of storage conditions on BA accumulation and the proliferation of biogenic amine-producing bacteria.

## 4. Conclusions

The study reported on bacterial communities and their relationship with the production of BAs in two typical fish products in China. The amounts of BAs were different among the fish species and regions. Both PUT and CAD were the most abundant BAs detected in fish product samples. All the microorganisms isolated from the two fish products produced multiple BAs. Corresponding to the BA contents in the samples, aminogenic bacteria mainly produced PUT and CAD. The strain in fermented mandarin fish with the largest bacterial count belonged to the species *Lactobacillus sakei* (mainly producing TYR and PUT), followed by *Bacillus cereus* (mainly producing PUT and HIS) and *Hafnia alvei* (mainly producing CAD, PUT, TYR and 2-PHE). The most abundant species producing BAs in semi-dried yellow croaker samples was *Shewanella baltica* (mainly producing PUT), followed by *Aeromonas veronii* (mainly producing CAD). *Photobacterium phosphoreum* produced the largest amount of HIS. The abundance, species and capability of BA-producing bacteria varied among different fish products. Further study is needed to focus on developing potential starter cultures for the reduction of BA accumulation, while preserving the sensory characteristics of the product.

## Figures and Tables

**Table 1:** The BA levels and microbial counts in fermented mandarin fish and semi-dried yellow croaker samples, expressed as means ± standard deviation (*n* = 3).

Fish Product	Sample No.	Region	mg/kg							Log CFU/g
			CAD	TYR	TRY	HIS	PUT	2-PHE	Total BAs	PCA
Fermented mandarin fish	1	Shanghai	36.90 ± 2.55 ^a^	ND	ND	8.28 ± 0.18 ^a^	56.84 ± 5.35 ^a^	0.36 ± 0.02 ^a^	102.38 ± 8.10 ^a^	4.9 ± 0.1 ^a^
	2	Huangshan	29.66 ± 1.87 ^b^	15.89 ± 1.00 ^a^	ND	6.94 ± 0.36 ^b^	49.97 ± 3.22 ^a^	0.21 ± 0.02 ^b^	102.67 ± 6.47 ^a^	5.3 ± 0.1 ^b^
Semi-dried yellow croaker	3	Ningde	3.94 ± 0.15 ^c^	ND	ND	1.82 ± 0.06 ^c^	4.17 ± 0.11 ^b^	ND	9.93 ± 0.32 ^b^	3.2 ± 0.1 ^c^
	4	Zhoushan	2.23 ± 0.14 ^d^	1.67 ± 0.08 ^b^	0.91 ± 0.05	1.68 ± 0.07 ^c^	ND	0.66 ± 0.02 ^c^	7.15 ± 0.36 ^c^	3.6 ± 0.1 ^d^

CAD—cadaverine; TYR—tyramine; TRY—tryptamine; HIS—histamine; PUT—putrescine; 2-PHE—2-phenylethylamine; PCA—plate count agar. Colony forming units (CFUs) were calculated based on plates containing 30 to 300 colonies each. Values in the same columns with different superscript letters (a, b, c or d) are significantly different (*p* < 0.05). ND—not detected.

**Table 2:** Diversity indices and coverage of fish samples from four different regions.

Samples from Different Regions	Sample No.	Region	Number	OTUs	Shannon	Chao	Ace	Simpson	Shannoneven	Coverage
Fermented mandarin fish	1	Shanghai	111,634 ^a^	304 ^a^	2.43 ^a^	313.63 ^a^	317.93 ^a^	0.26 ^a^	0.42 ^a^	0.9998 ^a^
	2	Huangshan	87,888 ^b^	432 ^ac^	2.51 ^a^	440.25 ^ac^	455.11 ^ac^	0.19 ^a^	0.41 ^a^	0.9995 ^a^
Semi-dried yellow croaker	3	Ningde	201,288 ^c^	581 ^bc^	3.53 ^a^	587.12 ^bc^	596.30 ^bc^	0.08 ^b^	0.55 ^a^	0.9998 ^a^
	4	Zhoushan	44,199 ^d^	542 ^bc^	3.52 ^a^	560.33 ^bc^	575.38 ^bc^	0.07 ^b^	0.56 ^a^	0.9985 ^a^

Values in same column with different superscript letters (a, b, c or d) are significantly different (*p* < 0.05).

**Table 3:** Species identification of BA-producing bacteria isolated from the two fish products by 16S rRNA gene sequencing.

Fish Product	Sample No.	Region	Isolates No. (*n* = 33)	Species	Identity (%)
Fermented mandarin fish	1	Shanghai	S1, S2	*Lactobacillus sakei*	99.76
			S3, S4	*Bacillus cereus*	98.85
			S5	*Hafnia alvei*	100.00
			S6	*Psychrobacter faecalis*	97.89
			S7	*Vagococcus fluvialis*	99.90
	2	Huangshan	H1, H2	*Lactobacillus sakei*	99.97
			H3, H4	*Hafnia alvei*	100.00
			H5	*Bacillus cereus*	99.65
			H6	*Psychrobacter faecalis*	99.51
			H7	*Aeromonas veronii*	99.97
			H8	*Staphylococcus caprae*	99.73
			H9	*Lactococcus garvieae*	98.98
Semi-dried yellow croaker	3	Ningde	N1, N2, N3	*Shewanella baltica*	98.66
			N4	*Ralstonia pickettii*	100.00
			N5	*Aeromonas veronii*	99.68
			N6	*Acinetobacter johnsonii*	99.55
			N7	*Psychrobacter faecalis*	100.00
	4	Zhoushan	Z1, Z2, Z3	*Shewanella baltica*	99.97
			Z4, Z5	*Aeromonas veronii*	99.98
			Z6	*Streptococcus parauberis*	97.16
			Z7	*Vibrio aphrogenes*	98.94
			Z8	*Photobacterium phosphoreum*	98.97
			Z9	*Prevotella 9* sp.	99.69
			Z10	*Aliivibrio salmonicida*	99.72

## Data Availability

The original contributions presented in this study are included in the article. Further inquiries can be directed to the corresponding author.

## References

[1] Mao J., Fu J., Zhu Z., Cao Z., Zhang M., Yuan Y., Chai T., Chen Y. (2023). Flavor characteristics of semi-dried yellow croaker (*Pseudosciaena crocea*) with KCl and ultrasound under sodium-reduced conditions before and after low temperature vacuum heating. Food Chem..

[2] Wang Y., Shen Y., Wu Y., Li C., Li L., Zhao Y., Hu X., Wei Y., Huang H. (2021). Comparison of the microbial community and flavor compounds in fermented mandarin fish (*Siniperca chuatsi*): Three typical types of Chinese fermented mandarin fish products. Food Res. Int..

[3] Gao X., Li C., He R., Zhang Y., Wang B., Zhang Z., Ho C. (2023). Research advances on biogenic amines in traditional fermented foods: Emphasis on formation mechanism, detection and control methods. Food Chem..

[4] Jaguey-Hernández Y., Aguilar-Arteaga K., Ojeda-Ramirez D., Añorve-Morga J., González-Olivares L.G., Castañeda-Ovando A. (2021). Biogenic amines levels in food processing: Efforts for their control in foodstuffs. Food Res. Int..

[5] FDA (2020). FDA and EPA safety levels in regulations and guidance. Department of Health and Human Services.

[6] Tian X., Gao P., Xu Y., Xia W., Jiang Q. (2021). Reduction of biogenic amines accumulation with improved flavor of low-salt fermented bream (*Parabramis pekinensis*) by two-stage fermentation with different temperature. Food Biosci..

[7] Dabadé D.S., Jacxsens L., Miclotte L., Abatih E., Devlieghere F., De Meulenaer B. (2021). Survey of multiple biogenic amines and correlation to microbiological quality and free amino acids in foods. Food Control.

[8] Yang Z., Liu S., Lv J., Sun Z., Xu W., Ji C., Liang H., Li S., Yu C., Lin X. (2020). Microbial succession and the changes of flavor and aroma in *Chouguiyu*, a traditional Chinese fermented fish. Food Biosci..

[9] Halász A., Baráth Á., Simon-Sarkadi L., Holzapfel W. (1994). Biogenic amines and their production by microorganisms in food. Trends Food Sci. Technol..

[10] Zhang X., Fang C., Huang D., Yang G., Tang Y., Shi Y., Kong K., Cao P., Cai Y. (2021). Determination of 8 biogenic amines in aquatic products and their derived products by high-performance liquid chromatography-tandem mass spectrometry without derivatization. Food Chem..

[11] Deng H., Wu G., Zhou L., Chen X., Guo L., Luo S., Yin Q. (2024). Microbial contribution to 14 biogenic amines accumulation in refrigerated raw and deep-fried hairtails (*Trichiurus lepturus*). Food Chem..

[12] Wang H., Luo Y., Huang H., Xu Q. (2014). Microbial succession of grass carp (*Ctenopharyngodon idellus*) filets during storage at 4°C and its contribution to biogenic amines’ formation. Int. J. Food Microbiol..

[13] Zhao Y., Wang Y., Li C., Li L., Yang L., Wu Y., Chen S., Zhao Y. (2021). Novel insight into physicochemical and flavor formation in naturally fermented tilapia sausage based on microbial metabolic network. Food Res. Int..

[14] Aregbe A.Y., Mu T., Sun H. (2019). Effect of different pretreatment on the microbial diversity of fermented potato revealed by high-throughput sequencing. Food Chem..

[15] Zhao C., Eun J. (2020). Shotgun metagenomics approach reveals the bacterial community and metabolic pathways in commercial hongeo product, a traditional Korean fermented skate product. Food Res. Int..

[16] Lin Y.T., Whitman W.B., Coleman D.C., Jin S.H., Wang H.C., Chiu C.Y. (2021). Soil bacterial communities at the treeline in subtropical alpine areas. Catena.

[17] Bhutia M.O., Thapa N., Shangpliang H.N.J., Tamang J.P. (2021). Metataxonomic profiling of bacterial communities and their predictive functional profiles in traditionally preserved meat products of Sikkim state in India. Food Res. Int..

[18] Bhutia M.O., Thapa N., Shangpliang H.N.J., Tamang J.P. (2020). High-throughput sequence analysis of bacterial communities and their predictive functionalities in traditionally preserved fish products of Sikkim, India. Food Res. Int..

[19] Ding T., Li Y. (2024). Biogenic amines are important indices for characterizing the freshness and hygienic quality of aquatic products: A review. LWT–Food Sci. Technol..

[20] Lou X., Zhai D., Yang H. (2021). Changes of metabolite profiles of fish models inoculated with *Shewanella baltica* during spoilage. Food Control.

[21] Dalgaard P., Madsen H.L., Samieian N., Emborg J. (2006). Biogenic amine formation and microbial spoilage in chilled garfish (Belone belone belone)–effect of modified atmosphere packaging and previous frozen storage. J. Appl. Microbiol..

[22] Belleggia L., Osimani A. (2023). Fermented fish and fermented fish-based products, an ever-growing source of microbial diversity: A literature review. Food Res. Int..

[23] Li C., Han X., Han B., Deng H., Wu T., Zhao X., Huang W., Zhan J., You L. (2023). Survey of the biogenic amines in craft beer from the Chinese market and the analysis of the formation regularity during beer fermentation. Food Chem..

[24] Saticioglu I.B., Yardimci B., Altun S., Duman M. (2021). A comprehensive perspective on a *Vagococcus salmoninarum* outbreak in rainbow trout broodstock. Aquaculture.

[25] Henríquez-Aedo K., Durán D., Garcia A., Hengst M.B., Aranda M. (2016). Identification of biogenic amines-producing lactic acid bacteria isolated from spontaneous malolactic fermentation of chilean red wines. LWT–Food Sci. Technol..

[26] Ding A., Zhu M., Qian X., Li J., Xiong G., Shi L., Wu W., Wang L. (2024). Identification of amine-producing bacterium (APB) in Chinese fermented mandarin fish (*Chouguiyu*) by high-throughput sequencing and culture-dependent methods. J. Food Compos. Anal..

[27] Li B., Lu S. (2020). The importance of amine-degrading enzymes on the biogenic amine degradation in fermented foods: A review. Process Biochem..

[28] Alvarez M.A., Moreno-Arribas M.V. (2014). The problem of biogenic amines in fermented foods and the use of potential biogenic amine-degrading microorganisms as a solution. Trends Food Sci. Technol..

[29] Meng J., Yang Q., Wan W., Zhu Q., Zeng X. (2022). Physicochemical properties and adaptability of amine-producing *Enterobacteriaceae* isolated from traditional Chinese fermented fish (*Suan yu*). Food Chem..

[30] Lavizzari T., Breccia M., Bover-Cid S., Vidal-Carou M.C., Veciana-Nogués M.T. (2010). Histamine, cadaverine, and putrescine produced in vitro by *Enterobacteriaceae* and *Pseudomonadaceae* isolated from spinach. J. Food Prot..

[31] Li X., Wang Y., Zhang G., Bi J., Hao H., Hou H. (2025). The role and the determination of the *LuxI* protein binding targets in the formation of biogenic amines in *Hafnia alvei* H4. Int. J. Food Microbiol..

[32] Benkerroum N. (2016). Biogenic amines in dairy products: Origin, incidence, and control means. Compr. Rev. Food Sci. Food Saf..

[33] Zhang Y., Zhang J., Lin X., Liang H., Zhang S., Ji C. (2022). *Lactobacillus* strains inhibit biogenic amine formation in salted mackerel (*Scomberomorus niphonius*). LWT–Food Sci. Technol..

[34] Wunderlichová L., Buňková L., Koutný M., Jančová P., Buňka B. (2014). Formation, Degradation, and Detoxification of Putrescine by Foodborne Bacteria: A Review. Compr. Rev. Food Sci. Food Saf..

[35] Lee S.G., Chang H.C. (2017). Assessment of Bacillus subtilis SN7 as a starter culture for *Cheonggukjang*, a Korean traditional fermented soybean food, and its capability to control *Bacillus cereus* in *Cheonggukjang*. Food Control.

[36] Shan K., Yao Y., Wang J., Zhou T., Zeng X., Zhang M., Ke W., He H., Li C. (2023). Effect of probiotic *Bacillus cereus* DM423 on the flavor formation of fermented sausage. Food Res. Int..

[37] Lerfall J., Shumilina E., Jakobsen A.N. (2022). The significance of *Shewanella* sp. strain HSO12, *Photobacterium phosphoreum* strain HS254 and packaging gas composition in quality deterioration of fresh saithe fillets. LWT–Food Sci. Technol..

[38] Lou X., Hai Y., Le Y., Ran X., Yang H. (2023). Metabolic and enzymatic changes of *Shewanella baltica* in golden pomfret broths during spoilage. Food Control.

[39] Bao X., Wang F., Yang R., Zhang Y., Fu L., Wang Y. (2021). Ornithine Decarboxylation System of *Shewanella baltica* Regulates Putrescine Production and Acid Resistance. J. Food Prot..

[40] Bjornsdottir-Butler K., May S., Hayes M., Abraham A., Benner R.A. (2020). Characterization of a novel enzyme from *Photobacterium phosphoreum* with histidine decarboxylase activity. Int. J. Food Microbiol..

[41] Bjornsdottir-Butler K., Abraham A., Harper A., Dunlap P.V., Benner R.A. (2018). Biogenic amine production by and phylogenetic analysis of 23 *Photobacterium* species. J. Food Prot..

[42] Kämpfer P., Batt C.A., Tortorello M.L. (2014). Acinetobacter. Encyclopedia of Food Microbiology.

[43] Guan T., Fu S., Wu X., Yu H., Liu Y. (2024). Bioturbation effect of artificial inoculation on the flavor metabolites and bacterial communities in the Chinese Mao-tofu fermentation. Food Chem. X.

